# The Renin-Angiotensin-Aldosterone System in Metabolic Diseases and Other Pathologies

**DOI:** 10.3390/ijms24087413

**Published:** 2023-04-18

**Authors:** Rudy M. Ortiz, Ryousuke Satou, Jia L. Zhuo, Akira Nishiyama

**Affiliations:** 1 Department of Molecular & Cell Biology, School of Natural Sciences, University of California, Merced, CA 95343, USA; 2 Department of Physiology and The Hypertension & Renal Center of Excellence, Tulane University School of Medicine, New Orleans, LA 70112, USA; rsato@tulane.edu (R.S.); jzhuo@tulane.edu (J.L.Z.); 3 Department of Pharmacology, Kagawa University Medical School, Kagawa 761-0793, Japan; nishiyama.akira@kagawa-u.ac.jp

It has been our pleasure to have been able to develop two special issues within the International Journal of Molecular Sciences: **(1)** Renin-Angiotensin-Aldosterone System in Pathologies and **(2)** Renin-Angiotensin-Aldosterone System in Metabolism & Disease. The planning for the first special issue started just before the height of the pandemic, which helped to bring the significance of the Renin-Angiotensin-Aldosterone System (RAAS) in disease to the forefront during the pandemic. Independent of the recent pandemic, angiotensin II (Ang II) has long been well-recognized for contributing to substrate metabolism [1,2,3,4] primarily through sympathetic nerve activity [5,6]. These initial references demonstrate the potential significance of the RAAS to the regulation of basic (non-pathological) biological processes. By the turn of the century, RAAS disruptors were demonstrating benefits on substrate metabolism independent of their anti-hypertensive effects (well reviewed in 10). Thus, our special issues are dedicated to helping advance the importance of studying RAAS and RAAS disruption in basic biological and pathological processes. Our first special issue on the RAAS and pathologies includes 7 papers (4 reviews and 3 empirical studies), which illustrate the diversity of the RAAS in biomedical conditions.

## 1. Review: Ang II and PNS—Novel Neural Insights

Much of the earlier work on Ang II and substrate metabolism demonstrated regulation through sympathetic nervous system [5,6]. The review article by Shanks and Ramchandra provides significant, complementary insights to the contributions of Ang II to the regulation of hypertension and cardiovascular hemodynamics via activation of the parasympathetic nervous system (PNS) [7]. This intriguing mechanism is based on data demonstrating substantial expression of the Ang II receptor type I (AT_1_) within the PNS. This important review also provides unique insights on the potential contributions of AT_2_ in the regulation of PNS activity. The implications include the need for future studies to have greater consideration of PNS in the regulation of Ang II-mediated processes in pathophysiological studies.

## 2. Review: Alternative RAS & Hypoxia

As previously mentioned, the COVID pandemic helped bring RAAS mechanisms to the forefront of scientific inquiry because of the interactions between angiotensin converting enzyme 2 (ACE2) and the COVID virus. The review by Rajtik et al. helps to simplify the complex mechanisms that implicates the alternative Renin-Angiotensin System (RAS), or the ACE2-Ang 1-7-Mas receptor pathway, in hypoxic conditions that can ultimately lead to cardiovascular and pulmonary complications especially during COVID [8]. Rajtik et al. provide novel insights on the complex mechanisms linking RAS and alternate RAS pathways to hypoxia-associated insults including perspectives on the duration of stimuli and causes in the heart and lung with an emphasis on clinical relevance as well as novel insights on future strategies to best study alternative RAS pathways.

## 3. Review: AT_2_ & Natriuresis in Hypertension

The contributions of AT_1_-mediated mechanisms to impaired natriuresis and the resulting elevation in arterial pressure are well-recognized and remain a significant area of research; however, the important review by Carey et al. illustrates the biological significance of AT_2_-mediated signaling in promoting natriuresis and potentially ameliorating the hypertension [9]. Carey et al. describe the potential cellular signaling pathway by which the activation of AT_2_ contributes to reduced proximal tubule Na^+^ reabsorption to promote natriuresis. The authors conclude that AT_2_ agonists may serve as excellent candidates for pharmacological intervention to promote natriuresis and help ameliorate volume-dependent hypertension.

## 4. Review: AT_1_ & Proximal Tubule Na^+^ in Hypertension

The intratubular renin-angiotensin system via AT_1a_ in the proximal tubule of the kidney is long recognized to contribute to maintaining basal and blood pressure homeostasis. However, its contributions to the development of hypertension and the underlying mechanisms remains incompletely understood. In this special issue, Leite et al. review recent developments and progress in this research field and provide new insights into the contributions of the Ang II-AT_1a_-Na^+^/H^+^ exchanger 3 (NHE3) axis in maintaining basal blood pressure homeostasis and the development of Ang II-dependent hypertension [10]. The deployment of the gold standard approach of *Cre/LoxP* allows for determining gain or loss of function of AT_1a_ and/or its downstream target, NHE3, in the proximal tubules. These new studies have consistently demonstrated that deletion of AT_1a_ or NHE3 selectively in the proximal tubules of the kidney lowers basal blood pressure, increases the pressure-natriuresis response, and induces natriuretic responses, whereas overexpression of an intracellular Ang II fusion protein or AT_1a_ selectively in the proximal tubules promote the opposite effects. This review highlights novel mechanisms by which the Ang II-AT_1_-NH3 axis in the proximal tubules contributes to the homeostatic control of arterial pressure and the development of Ang II-induced hypertension.

## 5. Article: Immunosuppression in Ang II-Dependent Hypertension

Current research has focused on elucidating immune-mediated mechanisms underlying elevation of blood pressure. Since intrarenal Ang II is elevated in many forms of hypertension, RAS is acknowledged as a key target in clinical and biochemical studies. Recent studies demonstrated that macrophages and enhanced production of pro-inflammatory cytokines can be crucial mediators of the renal angiotensinogen (AGT) elevation in hypertension. However, effects of immunosuppression on intrarenal AGT upregulation and the development of kidney injury in Ang II-dependent hypertension have not been established. Satou et al. demonstrated that Ang II-induced upregulation of intrarenal AGT expression, proteinuria, mesangial expansion, and renal tubulointerstitial fibrosis were attenuated by immunosuppression using mycophenolate mofetil (MMF) [11]. Furthermore, MMF treatment attenuated the augmentation of intrarenal NLRP3 mRNA, a component of inflammasome. These findings suggest that an activated immune system increases intrarenal AGT production in Ang II-dependent hypertension, which leads to the development of hypertensive kidney diseases.

## 6. Article: New Generation Mineralocorticoid Receptor Blockade in Hypertension

Several studies have indicated that activation of the mineralocorticoid receptor (MR) promotes the development of salt-dependent hypertension, even in cases of low or normal serum aldosterone levels. However, non-specific side effects of steroidal MR antagonists remain a major concern. Esaxerenone, a third-generation, non-steroidal MR antagonist, has recently been developed and has been shown to effectively reduce blood pressure (BP) in hypertensive patients. The study conducted by Hattori et al. aimed to investigate the effects of esaxerenone on body sodium homeostasis and its association with changes in BP in Dahl salt-sensitive (DSS) hypertensive rats [12]. The results showed that chronic antagonism of MR reduced BP and restored the diurnal variation of BP from the non-dipper to dipper pattern. These effects were associated with a reduction in Na^+^ content in the skin, skinned carcass, and total body demonstrating the significance of the MR to regulating whole body Na^+^ homeostasis in the context of salt-dependent hypertension. These findings suggest that esaxerenone is an effective treatment for salt-dependent hypertension by normalizing total body Na^+^ homeostasis and improving the diurnal variation of BP. The non-steroidal nature of esaxerenone may reduce the risk of non-specific side effects compared to steroidal MR antagonists observed in the past.

## 7. Article: AT_1_ Blockade and Hepatic Antioxidation

The contribution of Ang II and overactivation of AT_1_ to increased oxidative injury is well established; however, the mechanisms remain elusive. The study by Godoy-Lugo et al. provides mechanistic insight to the potential for the chronic hyperglycemia associated with metabolic syndrome and early insulin resistance to impair proper redox balance [13]. Chronic treatment with an AT_1_ blocker improved redox balance in the liver associated with reduced triglyceride accumulation (akin to reduced fatty liver) suggesting that a close link between AT_1_-mediated signaling, redox imbalance, and Non-Alcoholic Fatty Liver Disease (NAFLD) exists. Additionally, chronic ARB treatment improved Nrf2 levels in response to a glucose challenge implicating impaired Nrf2 signaling in the promotion of NAFLD by oxidative injury.

## 8. Summary

This compilation of articles has provided significant insights to the novel perspectives and current advances of RAAS and pathologies. This suite of articles demonstrates the diversity of RAAS-associated signaling within disorders that includes the cardiovascular, immune, integumentary, nervous, and renal systems as well as metabolism. The review articles provide the potential for unique and innovative paths for future directions while the unique empirical studies describe novel signaling pathways or previously unrevealed mechanisms or effects associated with RAAS and pharmaceutical disruption of RAAS. We read about the importance of balancing: **(1)** AT_1_- along with AT_2_-mediated signaling in the study of hypertension, **(2)** the PNS along with SNS in the study of cardiovascular function and disease, and **(3)** the proximal with the distal tubule regulation of Na^+^ in the development of hypertension. Additionally, the continued study of blood pressure-independent benefits of RAAS disruption especially in metabolic and immunological processes that are associated with other diseases should continue to be a focused area of research. This compilation of articles clearly demonstrate the need for additional studies of RAAS in various pathologies and provide clues for unique and innovative possibilities for such studies.
